# Compact Localized States in Engineered Flat-Band ${\mathscr{P}}{\mathscr{T}}$ Metamaterials

**DOI:** 10.1038/s41598-019-41155-8

**Published:** 2019-03-20

**Authors:** N. Lazarides, G. P. Tsironis

**Affiliations:** 1 Department of Physics, University of Crete, P. O. Box 2208, 71003 Heraklion, Greece; 2 National University of Science and Technology “MISiS”, Leninsky Prospekt 4, Moscow, 119049 Russia

## Abstract

The conditions leading to flat dispersionless frequency bands in truly one-dimensional parity-time (${\mathscr{P}}{\mathscr{T}}$) symmetric metamaterials comprised of split‐ring resonators (SRRs) arranged in a binary pattern are obtained analytically. In this paradigmatic system, in which the SRRs are coupled through both electric and magnetic dipole-dipole forces, flat-bands may arise from tailoring its natural parameters (such as, e.g., the coupling coefficients between SRRs) and not from geometrical effects. For sets of parameters which values are tailored to flatten the upper band of the spectrum, the solution of the corresponding quadratic eigenvalue problem reveals the existence of compact, two-site localized eigenmodes. Numerical simulations confirm the existence and the dynamic stability of such modes, which can be formed through the evolution of single-site initial excitations without disorder or nonlinearity.

## Introduction

Considerable research effort has focused the last decades in the development and investigation of artificial structures such as *metamaterials*^1,2^ and *parity-time (*${\mathscr{P}}{\mathscr{T}}-$*) symmetric materials*^3^, which exhibit properties not available in natural materials. Inspired by Veselago’s ideas^4^, Pendry and his collaborators suggested using split-ring resonator (SRR) arrays^5^ and thin-wire arrays^6^ to achieve effectively negative dielectric permeability and diamagnetic permittivity, respectively, in overlapping frequency bands. The combination of these two subsystems into a single artificial structure results in a negative refractive index medium, whose first realization was made in the turn of the 21st century^7^. The ${\mathscr{P}}{\mathscr{T}}-$ symmetric materials originated from the ideas and notions of non-Hermitian Quantum Mechanics^8^, which were later transferred to optical lattices^9^ and electronic systems^10^. The application of these ideas in electronic circuits has provided easily accessible experimental configurations as well as a link to the electrical circuit picture of SRR-based metamaterials; the latter may acquire ${\mathscr{P}}{\mathscr{T}}$ symmetry which relies on balanced gain and loss^11,12^. Such ${\mathscr{P}}{\mathscr{T}}$ metamaterials (PTMMs) may serve as paradigmatic systems that exhibit *isolated flat (dispersionless) bands*. This is possible because the SRRs in an SRR-based PTMM are coupled together both electrically and magnetically through dipole-dipole forces^13–15^. That key-property, along with the arrangment of the SRRs in a binary pattern, allow for the flattenning of the upper band of the two-band frequency spectrum through tailoring the coupling coefficients between SRRs. Clearly, this kind of band-flattening *is not due to geometrical effects*, i.e., the particular lattice structure (one-dimensional binary lattice).

Flat energy bands have been observed long ago in the electronic band structure of semiconductor heterostructures^16^ and superconducting cuprates^17^. Flat-bands were considered in the past as a theoretical convenience useful for obtaining exact analytical solutions of ferromagnetism (flat-band ferromagnetism)^18^. Recently, the possibility for dispersionless (diffraction-free in optics) propagation and robust localization in flat-band (FB) systems has initiated intensive research on simple crystal structures such as Lieb lattices^19–22^ as well as Kagomé^23^, merged^24^, cross-stitch^25^, Stub^26^, honeycomb^27^, rhombic^28^, sawtooth^29^, and diamond^25,30^ lattices that allow for precise FB engineering, even in the presence of nonlinearity^30^ and/or disorder^30,31^. Flat-band engineering methods have been also applied in tetragonal lattices beyond the tight-binding picture^32^. Analytical and numerical studies of the spectrum and localization properties of Lieb, Kagomé, and Stub ribbons reveal that ${\mathscr{P}}{\mathscr{T}}$ symmetry, relying on gain and loss, does not destroy the flat band in the Lieb ribbon (while it destroys the flat-band in the Kagomé and Stub ribbons)^33^. A detailed account on artificial flat band systems and related experiments is given in a recent review article^34^. Furthermore, research on numerus diverse systems such as complex networks^35^, Weyl semimetal superconductors^36^, organometalic frameworks^37^, twisted bilayer graphene^38^, graphene grain boundary^39^, and photonic crystal waveguides^40^, has also revealed the existence of FBs in their spectrum. Photonic flat bands, which have been reviewed in ref.^41^ have been designed for slow light propagation^42,43^. The existence of one or more FBs in the spectrum of a particular system is typically associated with the emergence of compact localized eigenmodes. Recently, the first experimental observation of diffraction-free propagation of such FB modes has been reported in Lieb photonic lattices^44,45^, and later in rhombic^28^ and Kagomé^46^ photonic lattices, as well as in bipartite optomechanical lattices^47^.

Here, a truly one-dimensional (1D) PTMM model in which a complete and isolated FB arises from tailoring its parameters and *not from geometrical effects*, is presented. A condition for the existence of a FB is obtained analytically, which can be satisfied by realistic parameter sets. In the presence of a FB in the frequency spectrum, compact, two-site localized eigenmodes are found by solving the corresponding quadratic eigenvalue problem (QEP). The existence and dynamic stability of such modes is confirmed by numerical simulations.

## Results

### Modelling, Frequency Dispersion, and Flat-Band Condition

**Figure 1:**
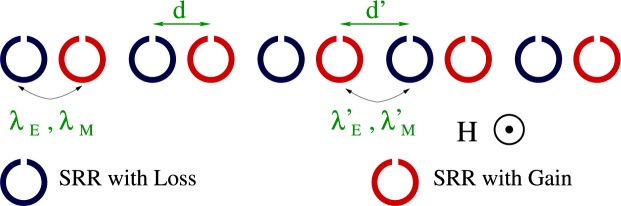
Schematic of a ${\mathscr{P}}{\mathscr{T}}$ metamaterial comprising split-ring resonators arranged in a one-dimensional binary pattern.

Consider a 1D array of SRRs arranged in a binary pattern as in Fig. 1, in which the SRRs have alternatingly loss (blue) and gain (red). In a balanced configuration, which is considered here, the amounts of loss and gain are equal. The SRRs shown in Fig. 1 (note the mutual orientation of their slits) interact both electrically and magnetically through dipole-dipole forces^14,15^, and can be regarded as *RLC* circuits, featuring a resistance *R*, an inductance *L*, and a capacitance *C*. Gain can be provided to an SRR by, e.g., mounting a negative resistance electronic device to its slit^11^. Using equivalent circuit models, the normalized equations governing the dynamics of the charge *q*_*n*_ stored in the capacitor *C* of the *n* th SRR are obtained as^11,48,49^

1 $${\lambda }_{M}^{^{\prime} }{\ddot{q}}_{2n}+{\ddot{q}}_{2n+1}+{\lambda }_{M}{\ddot{q}}_{2n+2}+\gamma {\dot{q}}_{2n+1}+{q}_{2n+1}=-\{{\lambda }_{E}^{^{\prime} }{q}_{2n}+{\lambda }_{E}{q}_{2n+2}\},$$

2 $${\lambda }_{M}{\ddot{q}}_{2n-1}+{\ddot{q}}_{2n}+{\lambda }_{M}^{^{\prime} }{\ddot{q}}_{2n+1}-\gamma {\dot{q}}_{2n}+{q}_{2n}=-\{{\lambda }_{E}{q}_{2n-1}+{\lambda }_{E}^{^{\prime} }{q}_{2n+1}\},$$

where *λ*_*E*_ and *λ*_*M*_ (${\lambda }_{E}^{^{\prime} }$ and ${\lambda }_{M}^{^{\prime} }$) are respectively the electric and magnetic coupling coefficients between SRRs with center-to-center distance *d* (*d*′), *γ* is the gain/loss coefficient (*γ* > 0), and the overdots denote derivation with respect to the normalized temporal variable $\tau ={\omega }_{LC}t$, with ${\omega }_{LC}^{-1}=\sqrt{LC}$.

By substituting the plane wave solution

3 $$\begin{array}{cc}{q}_{2n}=A\,\exp [i(2n\kappa -{\rm{\Omega }}\tau )], & {q}_{2n+1}=B\,\exp [i((2n+1)\kappa -{\rm{\Omega }}\tau )]\end{array}$$

into Eqs (1 and 2), where *κ* is the normalized wavevector and Ω is the frequency in units of *ω*_*LC*_, and requesting nontrivial solutions for the resulting stationary problem, we obtain

4 $${{\rm{\Omega }}}_{\kappa }^{2}=\frac{1}{2a}(-b\pm \sqrt{{b}^{2}-4ac}),$$

where

5 $$\begin{array}{rcl}a & = & 1-{({\lambda }_{M}^{2}+{\lambda }_{M}^{^{\prime} 2})}^{2}-2{\lambda }_{M}{\lambda }_{M}^{^{\prime} }\,\cos \,\mathrm{(2}\kappa \mathrm{).}\\ b & = & 2({\lambda }_{E}{\lambda }_{M}+{\lambda }_{E}^{^{\prime} }{\lambda }_{M}^{^{\prime} })+2({\lambda }_{E}{\lambda }_{M}^{^{\prime} }+{\lambda }_{E}^{^{\prime} }{\lambda }_{M})\cos \,\mathrm{(2}\kappa )-2+{\gamma }^{2},\\ c & = & 1-{({\lambda }_{E}^{2}+{\lambda }_{E^{\prime} }^{2})}^{2}-2{\lambda }_{E}{\lambda }_{E}^{^{\prime} }\,\cos (2\kappa ).\end{array}$$

In the exact ${\mathscr{P}}{\mathscr{T}}$ phase, Eq. (4) gives a gapped spectrum with two frequency bands separated by a gap. The FB condition is obtained by requesting $d({{\rm{\Omega }}}_{\kappa }^{2})/d\kappa =0$ for any *κ* in the first Brillouin zone. After tedious calculations, we get

6 $$[\frac{1}{ {\mathcal R} ^{\prime} }(1-{\lambda }_{M}^{2})+ {\mathcal R} +{\mathscr{G}}^{\prime} (1-{\lambda }_{E}^{2})][\frac{1}{ {\mathcal R} }(1-{\lambda }_{M}^{^{\prime} 2})+{\mathscr{G}}^{\prime} + {\mathcal R} (1-{\lambda }_{E}^{^{\prime} 2})]=\mathrm{0,}$$

where

7 $$\begin{array}{cccc} {\mathcal R} =\frac{{\lambda }_{M}}{{\lambda }_{E}}, &  {\mathcal R} ^{\prime} =\frac{{\lambda }_{M}^{^{\prime} }}{{\lambda }_{E}^{^{\prime} }}, & {\mathscr{G}}=-\,2+{\gamma }^{2}+2{\lambda }_{E}{\lambda }_{M} & {\mathscr{G}}^{\prime} =-\,2+{\gamma }^{2}+2{\lambda }_{E}^{^{\prime} }{\lambda }_{M}^{^{\prime} }\mathrm{.}\end{array}$$

**Figure 2:**
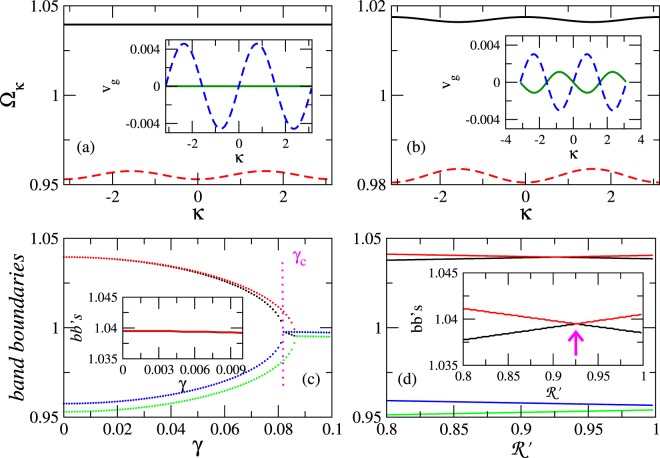
(**a**) Frequency bands Ω_*κ*_(*κ*) for a ${\mathscr{P}}{\mathscr{T}}$ metamaterial with *γ* = 0.003, *λ*_*E*_ = −0.123952, *λ*_*M*_ = −0.040128, ${\lambda }_{E}^{^{\prime} }$ = −0.027, and ${\mathcal R} ^{\prime} =0.92547$. The upper band at ${{\rm{\Omega }}}_{\kappa }\sim 1.03949$ (black-solid curve) is flat. Inset: The corresponding group velocities *v*_*g*_ (=0 for the flat band). (**b**) Same as in (**a**) for *γ* = 0.003, *λ*_*E*_ = −0.055, *λ*_*M*_ = −0.02, ${\lambda }_{E}^{^{\prime} }$ = −0.027, and ${\mathcal R} ^{\prime} =0.92547$. (**c**) The band-boundaries as a function of *γ* for the coupling coefficients in (**a**). The vertical segment indicates *γ* = *γ*_*c*_. Inset: Enlargement of the upper band-boundaries for low *γ*. (**d**) The band-boundaries as a function of ${\mathcal R} ^{\prime}$, with *γ*, *λ*_*E*_, *λ*′_*E*_, and *λ*_*M*_ as in (**a**). Inset: Enlargement of the boundaries of the upper band around the value of ${\mathcal R} ^{\prime}$ for which the band is flat (shown in (**a**)).

The values of ${\mathcal R} ^{\prime}$, *λ*_*E*_, *λ*_*M*_, and *γ*, for which the expression in the first squared brackets in Eq. (6) equals to zero, provide a physically acceptable parameter set which flattens the upper band of the spectrum over the whole Brillouin zone. For such a parameter set, the calculated frequency spectrum (from Eq. (4)) contains a *completely flat, isolated upper band* (Fig. 2(a)) with zero group velocity *v*_*g*_ for any *κ* in the first Brillouin zone (inset).

The electric and magnetic coupling coefficients between single SRRs depend crucially on their mutual position. In the model adopted here, the SRRs assume planar geometry; even in this case, however, the coupling coefficients depend strongly on the mutual orientation of the gaps. For the configuration of Fig. 1, these coefficients have been calculated accurately and plotted in Fig. 2(a) of ref.^15^ using the approach presented in ref.^50^, where the effect of retardation has been also taken into account. In these works, single SRRs having typical dimensions have been considered, while the authors have verified that the resonance frequencies of pairs of SRRs coupled with the calculated coefficients match the resonances found by direct numerical simulations using commercial software (CST Microwave Studio). This indicates that the calculated coupling coefficients can quantitatively describe the near-field interaction between SRRs. According to ref.^15^, the coupling coefficients used in Fig. 2(a) correspond to distances *d*∼2 and *d*′∼3.7 between neighboring SRRs in units of their radii, that seems in principle feasible. On the contrary, for parameter sets not satisfying Eq. (6), non-flat bands with *κ*− dependent group velocities *v*_*g*_ such as those shown in Fig. 2(b) are typically obtained. The band-boundaries (BBs), i.e., the extremal frequencies in each band, are shown as a function of the gain/loss coefficient *γ* in Fig. 2(c). A critical value of that coefficint, *γ* = *γ*_*c*_, separates the exact or unbroken from the broken ${\mathscr{P}}{\mathscr{T}}$ phase. For *γ* < *γ*_*c*_ (exact or unbroken ${\mathscr{P}}{\mathscr{T}}$ phase), the BBs of the upper band (red and black dotted curves) practically coincide for a substantial interval of *γ* indicating zero bandwidth (i.e., a FB), while the width of the lower band (limited by the blue and green dotted curves) remains almost constant up to *γ* = *γ*_*c*_. The flatness of the upper band for low *γ* can be seen more clearly in the inset. In Fig. 2(d), the BBs are plotted as a function of ${\mathcal R} ^{\prime}$; the width of the upper band (limited by the red and black solid curves) consecutively decreases, goes through zero at a critical value of ${\mathcal R} ^{\prime}$ indicated by the arrow, and then increases with increasing ${\mathcal R} ^{\prime}$ (see also the inset). At that critical value of ${\mathcal R} ^{\prime}$ the two bands coincide with those shown in Fig. 2(a).

### The Quadratic Eigenvalue Problem

Eqs (1 and 2) can be written in matrix form as

8 $$\hat{{\bf{M}}}\ddot{{\bf{Q}}}={\hat{{\bf{C}}}}_{1}\dot{{\bf{Q}}}+\hat{{\bf{K}}}Q,$$

where ${\bf{Q}}={[{q}_{1}{q}_{2}\cdots {q}_{N}]}^{T}$ is an *N*− dimensional vector, with *N* being the total number of SRRs, and the *N* × *N* matrices $\hat{{\bf{M}}}$ and $\hat{{\bf{K}}}$ are given by

9 $$\hat{{\bf{M}}}=[\begin{array}{cccccccc}1 & {\lambda }_{M} & 0 & 0 & 0 & 0 & 0 & \ldots \\ {\lambda }_{M} & 1 & {\lambda }_{M}^{^{\prime} } & 0 & 0 & 0 & 0 & \ldots \\ 0 & {\lambda }_{M}^{^{\prime} } & 1 & {\lambda }_{M} & 0 & 0 & 0 & \ldots \\ 0 & 0 & {\lambda }_{M} & 1 & {\lambda }_{M}^{^{\prime} } & 0 & 0 & \ldots \\ 0 & 0 & 0 & {\lambda }_{M}^{^{\prime} } & 1 & {\lambda }_{M} & 0 & \ldots \\ 0 & 0 & 0 & 0 & {\lambda }_{M} & 1 & {\lambda }_{M}^{^{\prime} } & \ldots \\ 0 & 0 & 0 & 0 & 0 & {\lambda }_{M}^{^{\prime} } & 1 & \ldots \\ \vdots  & \vdots  & \vdots  & \vdots  & \vdots  & \vdots  & \vdots  & \ddots \end{array}],\hat{{\bf{K}}}=(\,-\,\mathrm{1)}[\begin{array}{cccccccc}1 & {\lambda }_{E} & 0 & 0 & 0 & 0 & 0 & \ldots \\ {\lambda }_{E} & 1 & {\lambda }_{E^{\prime} } & 0 & 0 & 0 & 0 & \ldots \\ 0 & {\lambda }_{E}^{^{\prime} } & 1 & {\lambda }_{E} & 0 & 0 & 0 & \ldots \\ 0 & 0 & {\lambda }_{E} & 1 & {\lambda }_{E}^{^{\prime} } & 0 & 0 & \ldots \\ 0 & 0 & 0 & {\lambda }_{E}^{^{\prime} } & 1 & {\lambda }_{E} & 0 & \ldots \\ 0 & 0 & 0 & 0 & {\lambda }_{E} & 1 & {\lambda }_{E}^{^{\prime} } & \ldots \\ 0 & 0 & 0 & 0 & 0 & {\lambda }_{E}^{^{\prime} } & 1 & \ldots \\ \vdots  & \vdots  & \vdots  & \vdots  & \vdots  & \vdots  & \vdots  & \ddots \end{array}]$$

**Figure 3:**
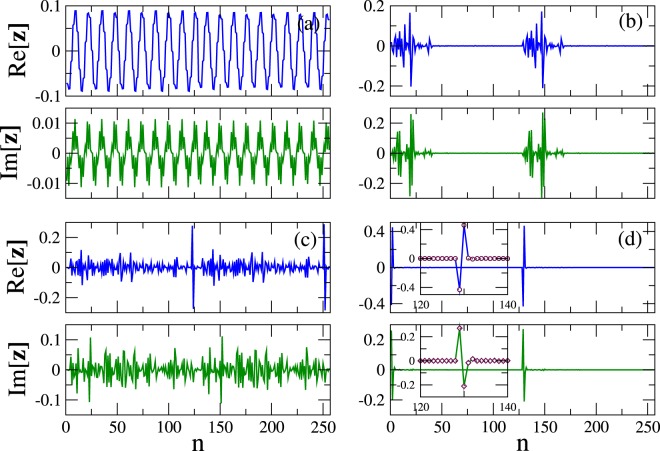
Real (blue) and imaginary (green) parts of four eigenmodes obtained by solving the quadratic eigenvalue problem Eq. (11) for *N* = 128, *γ* = 0.01, *λ*_*E*_ = −0.1200046, *λ*_*M*_ = −0.0400493, ${\lambda }_{E}^{^{\prime} }$ = −0.027, and ${\mathcal R} ^{\prime} =0.9291948$. The eigenfrequency of the flat band is ${{\rm{\Omega }}}_{FB}\simeq 1.03740$. (**a**) An extended eigenmode from the lower (non-flat) band. (**b**) A partially localized flat-band eigenmode. (**c**) A highly localized flat-band eigenmode. (**d**) The compact, two-site localized flat-band eigenmode. Insets: Enlargements around the localization region.

The *N* × *N* matrix ${\hat{{\bf{C}}}}_{1}$ is diagonal, with ${({\hat{{\bf{C}}}}_{1})}_{n,n}=\gamma {(-\mathrm{1)}}^{n}$ (*n* = 1, …, *N*). By substituting ${\bf{Q}}={{\bf{Q}}}^{e}\,{e}^{i{\rm{\Omega }}\tau }$ into Eq. (8) we get

10 $$\{{{\rm{\Omega }}}^{2}\hat{{\bf{M}}}+{\rm{\Omega }}\hat{{\bf{C}}}+\hat{{\bf{K}}}\}{{\bf{Q}}}^{e}=\hat{{\bf{0}}},$$

where $\hat{{\bf{C}}}\equiv i{\hat{{\bf{C}}}}_{1}$. Eq. (10) is a QEP^51^ with Ω being the eigenvalue and **Q**^*e*^ the corresponding *N*− dimensional eigenvector. It can be solved by standard eigenproblem solvers after its *linearization by the classical augmentation procedure*^52,53^, which transforms square matrices of order *N* to 2*N*. Then, Eq. (10) is reduced to a standard eigenvalue problem (SEP)

11 $$\hat{{\bf{D}}}z={\rm{\Omega }}{\bf{z}},\,{\bf{z}}=[\begin{array}{c}{{\bf{Q}}}^{e}\\ {\rm{\Omega }}{{\bf{Q}}}^{e}\end{array}],\,\hat{{\bf{D}}}=[\begin{array}{cc}\hat{{\bf{0}}} & \hat{{\bf{I}}}\\ -{\hat{{\bf{M}}}}^{-1}\hat{{\bf{K}}} & -{\hat{{\bf{M}}}}^{-1}\hat{{\bf{C}}}\end{array}],$$

where $\hat{{\bf{I}}}$ is the *N* × *N* identity matrix and ${\hat{{\bf{M}}}}^{-1}$ the inverse of $\hat{{\bf{M}}}$. In what follows, *γ* < *γ*_*c*_ so that the system is in the exact (unbroken) ${\mathscr{P}}{\mathscr{T}}$ phase, and therefore all its eigenvalues are real. A few selected eigenmodes **Q**^*e*^ are shown in Fig. 3. The eigenmodes from the non-flat (lower) band are all extended, as that shown in Fig. 3(a). The other three eigenmodes in Fig. 3 belong to the flat (upper) band, and therefore they correspond to the same frequency eigenvalue Ω_*κ*_ = Ω_*FB*_. Figure 3(b–d) show a partially localized, a highly localized, and a compact two-site localized eigenmode, respectively. The insets in Fig. 3(d) enlarge the localization region. The existence of compact localized eigenvectors in a class of FB models has been also demonstrated in ref.^25^. In that work, a model with at least one FB was considered, whose eigenvectors in the Bloch representation may be mixed to obtain highly localized FB eigenvectors, due to macroscopic degeneracy. While there is not any general theorem which states that among all these combinations there will be compact localized eigenvectors, such eigenvectors do exist in several FB models. Compact localized states were constructed, which are actually exact FB localized eigenstates, and then classified according to the number of unit cells they occupy. Such compact localized states occupying one unit cell form a complete and orthogonal basis that makes possible to detangle them from the rest of the lattice. The inversion of the detangling procedure provides the most general FB generator having localized eigenstates that occupy one unit cell. Such models include cross-stich and diamond chains, both quasi-one dimensional at the single-site level. Here, a purely one-dimensional system is considered in the form of a ${\mathscr{P}}{\mathscr{T}}$ symmetric SRR chain arranged in a binary pattern, whose geometry does not in general provides a FB. However, that system does possess a FB when the coupling coefficients and the gain/loss factor satisfy the condition Eq. (6). In that case, the resulting QEP is directly solved after being transformed into a SEP, demonstrating the existence of a compact localized FB eigenstate as that shown in Fig. 3(d). In the light of these findings, it would be expected that the PTMM in the exact ${\mathscr{P}}{\mathscr{T}}$ phase supports compact localized excitations which in general could be expressed as linear superpositions of a small number of eigenmodes.

The existence of localized states or modes in discrete lattices has been investigated intensively in the past. It has now been established that it may be due to quenched disorder (random lattices), a phenomenon based on wave interference which is known as Anderson localization^54^. In the presence of randomly distributed impurities in a metal, for example, different paths taken by an electron can interfere destructively, leading to localization of its wavefunction. The concept of Anderson localization is applicable to, and has actually been observed experimentally in a variety of physical systems. Localization may also appear in nonlinear lattices which are perfectly periodic; this effect is known as intrinsic localization, which leads to localized states of the discrete breather type^55^. Discrete breather excitations can be created in nonlinear lattices from an initially extended state through the standard modulational instability mechanism. Such localized states have been also experimentally observed in a variety of physical systems. Apart from the case of quenched disorder and nonlinearity, localized states can also arise in tight-binding (nearest-neighbor) models from particular lattice geometries in which destructive interference leads to the emergence of FBs. FB models typically possess localized eigenstates, although there is no general theorem to guarantee their existence. Since in the model considered here, neither disorder nor nonlinearity is present, the apearance of compact localized eigenstates for the PTMM is only due to the existence of the FB in its frequency spectrum.

### Numerical Simulations

**Figure 4:**
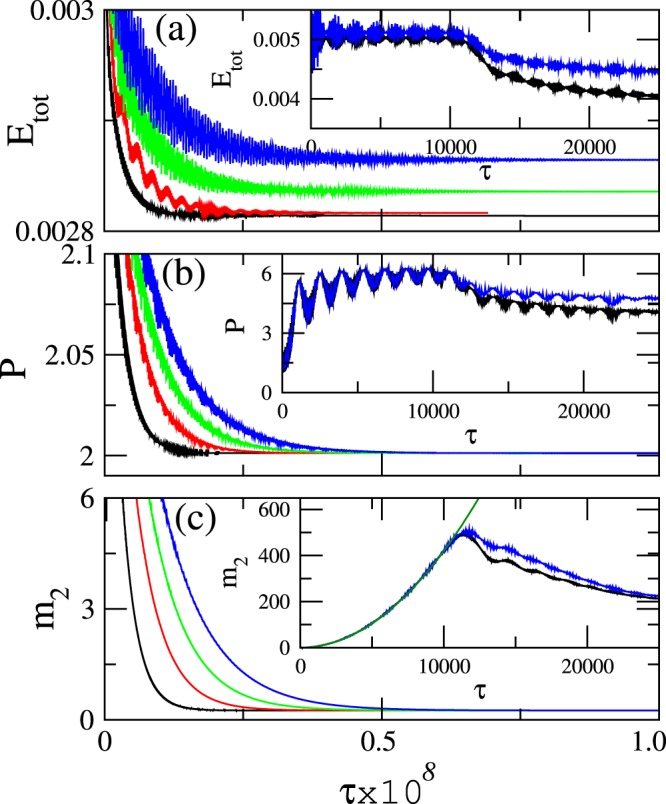
Total energy *E*_*tot*_ (**a**), energetic participation number *P* (**b**), and second moment *m*_2_ (**c**), as functions of *τ* for *N* = 128, *N*_*e*_ = 16, and *γ* = 0.003, *λ*_*E*_ = −0.123952, *λ*_*M*_ = −0.0400518, ${\mathcal R} ^{\prime} =0.92547$ (black); $\gamma =0.006$, ${\lambda }_{E}=-\,0.120021$, ${\lambda }_{M}=-\,0.0400518$, ${\mathcal R} ^{\prime} =0.9288096$ (red); $\gamma \mathrm{=0.009}$, ${\lambda }_{E}=-\,0.1200284$, ${\lambda }_{M}=-\,0.0399891$, ${\mathcal R} ^{\prime} =0.9290096$ (green); *γ* = 0.012, ${\lambda }_{E}=-\,0.120122$, ${\lambda }_{M}=-\,0.0400118$, ${\mathcal R} ^{\prime} =0.9293204$ (blue). Insets: The curves for *γ* = 0.003 (black) and 0.012 (blue) in a short time-scale. In the inset in (**c**), the curve 4.2 × 10^−6^*τ*^2^ (green) is also plotted.

Eqs (1 and 2), implemented with free-end boundary conditions ${q}_{0}(\tau )={q}_{N+1}(\tau )=0$, are integrated in time with a standard 4 th order Runge-Kutta algorithm. The initial conditions are single-site excitations of the form

12 $$\begin{array}{c}{q}_{n}(\tau =0)=A\,{\rm{for}}\,n=N\mathrm{/2}+1,\\ {q}_{n}(\tau =\mathrm{0)}=0\,{\rm{for}}\,n\ne N\mathrm{/2}+1,\\ {\dot{q}}_{n}(\tau =\mathrm{0)}=0\,{\rm{for}}\,n=\mathrm{1,}\ldots ,N,\end{array}$$

where typically *A* = 0.1. For *N*_*e*_ SRRs at each end of the PTMM, gain has been replaced by equal amount of loss, as if the ${\mathscr{P}}{\mathscr{T}}$ metamaterial were embedded into a lossy metamaterial. It is found empirically that this is the most effective way to stabilize localized modes in PTMMs, even in the presence of nonlinearity^11^ (in which case the localized modes are of the discrete breather type). The need for introducing these lossy parts at each end of the PTMM comes from the fact that the initial condition Eq. (12) is clearly not an exact eigenmode of Eqs (1 and 2). However, after long time-integration, that type of initial condition may relax to an exact eigenmode with a lower energy than the initial one. During this process, some of the initial energy spreads towards the ends of the PTMM, where it is dissipated. Thus, these lossy parts help the excess energy to go smoothly away during the transient phase of integration, and allow the formation of a stable compact localized FB state with constant energy. This gradual energy removal is clearly observed in Fig. 4(a) discussed below.

The total energy, the (energetic) participation number, and the second moment

13 $$\begin{array}{ccc}{E}_{tot}=\frac{1}{2}\{{\dot{{\bf{Q}}}}^{T}\dot{{\bf{Q}}}+{{\bf{Q}}}^{T}{\bf{Q}}+{{\bf{Q}}}^{T}\hat{K}{\bf{Q}}+{\dot{{\bf{Q}}}}^{T}M\dot{{\bf{Q}}}\}, & P=\frac{1}{\sum _{n}{{\boldsymbol{\varepsilon }}}_{n}^{2}}, & {m}_{2}=\sum _{n}{(n-\bar{n})}^{2}{{\boldsymbol{\varepsilon }}}_{n},\end{array}$$

respectively, where $\bar{n}={\sum }_{n}n\,{{\boldsymbol{\varepsilon }}}_{n}$ is the center of energy with ${{\boldsymbol{\varepsilon }}}_{n}={E}_{n}/{E}_{tot}$ being the energy density, are shown in Fig. 4 as functions of *τ*. The four curves are obtained for parameter sets which satisfy the FB condition; the coupling coefficients have very similar values, while *γ* increases from 0.03 to 0.12 in steps of 0.03. In all cases, a steady FB state is reached at the end of the integration time; however, the transient period is longer and *E*_*tot*_ is higher for higher *γ*. In the insets of Fig. 4(a–c), the quantities *E*_*tot*_, *P*, and *m*_2_, respectively, are plotted for the first 25,000 time units (t.u.). The energy *E*_*tot*_ strongly fluctuates, but it remains on average constant until *τ*∼12,000 t.u. At the same time, *P* increases while fluctuating strongly and reaches *P* = 6, while *m*_2_ increases ∝*τ*^2^ indicating ballistic spreading of the initial state. Around *τ*∼12,000 t.u., the energy which spreads out from the initial state has reached the lossy ends of the PTMM where it is dissipated. After that time, *E*_*tot*_, *P*, and *m*_2_ decrease gradually until they saturate to constant values with vanishingly small fluctuations, indicating that a steady state has been reached. The constancy of *m*_2_, in particular, indicates that energy spreading has been stopped. Part of the initial excitation remains localized on two SRRs, as indicated by the value of *P* = 2 which measures the number of the energetically strongest excited sites.

**Figure 5:**
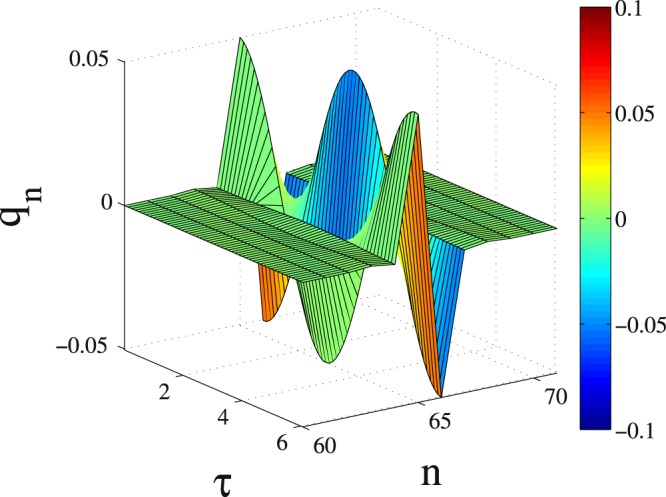
Spatiotemporal diagram of the charges *q*_*n*_ after ∼10^8^ time units of integration for $\gamma \,=\,0.01$, ${\lambda }_{E}=-\,0.1200046$, ${\lambda }_{M}=-\,0.0400493$, ${\mathcal R} ^{\prime} =0.9291948$, $N\,=\,128$, ${N}_{e}\mathrm{=16}$ (${{\rm{\Omega }}}_{FB}\simeq 1.03740$). Only part of the ${\mathscr{P}}{\mathscr{T}}$ metamaterial is shown for clarity.

**Figure 6:**
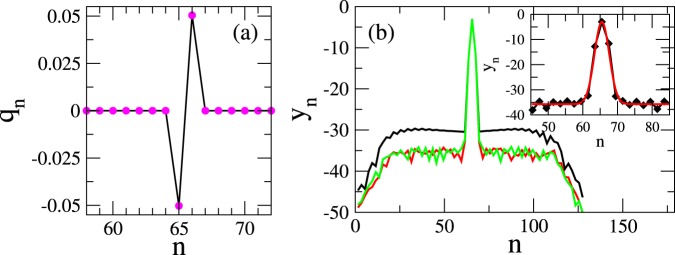
(**a**) Charge profile *q*_*n*_ at maximum oscillation amplitude. Parameters as in Fig. 5. (**b**) The function *y*_*n*_ after integration for *τ*_0_ = 1.5 × 10^6^ (black), 2 × 10^6^ (red), 3 × 10^6^ (green), time units. Inset: Fitting of *y*_*n*_ with an appropriate function (see text).

Inspection of the *q*_*n*_(*τ*) in Fig. 5 reveals that the excitation is localized to only two, neighboring SRRs. The charges stored in the capacitors of these SRRs oscillate in anti-phase with frequency Ω_*FB*_; charge oscillations in the rest of the SRRs seems to have vanishingly small amplitude (see also Fig. 6(a)). Note that if the FB condition Eq. (6) is not satisfied, the initial excitations disperse rapidly in the lattice without leaving behind any trace of localization. Compact localized excitations, which tails decay as a stretched exponential or superexponential, often appear in discrete systems with nonlinear dispersion^56^. Here, the compact localized states appear solely due to the FB in the absence of nonlinearity or disorder, and hence there are significant differences. In Fig. 6(b), the quantity ${y}_{n}=\,\mathrm{ln}[\frac{1}{2}(|{q}_{2n-1}|+|{q}_{2n}|)]$ is plotted as a function of *n*; the three curves correspond to different integration times, *τ*_0_. In obtaining the results in Fig. 6(b), convergence to a steady localized FB state was accelerated by continually eliminating the energy spreading away from the localization region during half of the integration time, i.e., for *τ*_0_/2 time units. During this time, we set ${q}_{n}={\dot{q}}_{n}=0$ for $n=1,...,N\mathrm{/2}-3$ and $n=N\mathrm{/2}+\mathrm{6,}\,\ldots ,\,N$, every 10 periods ${T}_{FB}=2\pi /{{\rm{\Omega }}}_{FB}$ of integration. This does not affect the region in which we expect the compact localized FB state to be formed, i.e., the eight sites for $n=\frac{N}{2}-\mathrm{2,}\,\frac{N}{2}-\mathrm{1,}\ldots ,\frac{N}{2}+\mathrm{4,}\,\frac{N}{2}+5$ (localization region). Note that for the chosen initial condition, the compact two-site FB localized state is generated at the sites $n=N\mathrm{/2}+1$ and $n=N\mathrm{/2}+2$, i.e., in the middle of the localization region. For $\tau  > {\tau }_{0}\mathrm{/2}$ the integration proceeds without elimination of the energy for *τ*_0_/2 more time units. The almost horizontal segments between $16\mathop{ < }\limits_{ \tilde {}}n\mathop{ < }\limits_{ \tilde {}}63$ and $66\mathop{ < }\limits_{ \tilde {}}n\mathop{ < }\limits_{ \tilde {}}112$ (i.e., those parts of the lattice which do not belong either to the lossy ends nor to the localization region) correpond to the tails of the localized FB states. With increasing *τ*_0_, the tails become more and more negative until they saturate at ${y}_{n}\sim -\,35$ (the limit of double precision arithmetics) at *τ*_0_ = 2 × 10^6^. In the inset, the *y*_*n*_ profile at *τ*_0_ = 3 × 10^6^ t.u. (black curve) is fitted by ${y}_{n}=b+({\alpha }_{0}-b){\rm{e}}{\rm{x}}{\rm{p}}[c{(n-{x}_{0})}^{2}]$ (red curve), where *x*_0_ = 65.5 and *α*_0_ = −2.9887 are taken from the numerical data. The fitting parameters are *b* = −35.684 and *c* = −0.109955. Using the procedure of energy elimination, the relaxation time towards the formation of a compact localized FB state is reduced considerably. For example, after 3 × 10^6^ time units of time-integration, the quantities *E*_*tot*_, *P*, and *m*_2_ take respectively the values 2.855 × 10^−3^, 2.005, and 2.826 × 10^−1^. In order to reach these values without energy elimination, the integration time needed is respectively ∼9.13 × 10^6^, ∼2.82 × 10^7^, and ∼4.62 × 10^7^ time units. Note that without energy elimination there are still significant fluctuations around the above values for *E*_*tot*_, *P*, and *m*_2_. Thus, the quantities *E*_*tot*_, *P*, and *m*_2_ do not seem to relax at the same rate when they are calculated with and without elimination of energy. However, as far as the localization region is concerned, a comparison of the relaxation times for $P\simeq 2.005$, i.e., 3 × 10^6^ and ∼2.82 × 10^7^, indicates a difference of an order of magnitude. Note that $P\simeq 2.005$ indicates that there are only two strongly excited sites in the PTMM.

## Conclusion

By tailoring the model parameters of a 1D PTMM comprising SRRs arranged in a binary pattern, an isolated and completely FB may appear in its frequency spectrum. The analytical condition, which those parameters have to satisfy in order to flatten the upper band, is obtained. The solution of the QEP reveals the existence of compact, localized FB eigenmodes, in which most of the energy is concentrated in two neighboring SRRs which are separated by distance *d*′. This is consistent with earlier results on nonlinear binary PTMMs, for which the fundamental discrete breathers are two-site ones (and not single-site ones)^11^. The formation of compact, two-site localized FB states from single-site initial excitations is numerically confirmed. Since the FB is isolated, these FB states could be continued into compact breather-like (nonlinear) excitations. Note that the possibility of flattening one of the bands of the spectrum is solely due to model parameter engineering, and not to any geometrical effects. In this aspect, the existence of two types of coupling between SRRs, i.e., electric and magnetic coupling, is crucial. Note also that similar results would have been obtained for *γ* = 0, in which case only three parameters, i.e., *λ*_*E*_, *λ*_*M*_, and ${\mathcal R} ^{\prime}$, should be matched to satisfy the corresponding FB condition. However, as it is demonstrated here, the band-flattening capability is not harmed by the ${\mathscr{P}}{\mathscr{T}}$ symmetry, as long as the PTMM is in the exact ${\mathscr{P}}{\mathscr{T}}$ phase.
